# Insights Into Endovascular Management of Superior Vena Cava Obstructions

**DOI:** 10.3389/fcvm.2021.765798

**Published:** 2021-11-24

**Authors:** Alexandre Ponti, Sarah Saltiel, David C. Rotzinger, Salah D. Qanadli

**Affiliations:** Department of Diagnostic Radiology and Interventional Radiology, Lausanne University Hospital (CHUV) and Univerity of Lausanne (UNIL), Lausanne, Switzerland

**Keywords:** superior vena cava, venous obstruction, venous disease, endovascular therapies, angioplasty, stent

## Abstract

Superior vena cava obstruction results from any limitation of blood flow through the superior vena cava. Circulation to the heart may persist through various collateral vessels whose development depends on the level of obstruction. Depending on the level and degree of occlusive disease, the severity of clinical symptoms may vary considerably, up to lethal. Etiologies have changed dramatically in recent years, mainly due to the increasing use of intravascular devices. However, guidelines for treatment are lacking, and various options are available. Endovascular therapies developed considerably in recent years, may offer a rapid improvement in symptoms and proved to be safe. However, knowledge and selection of appropriate techniques are essential to venous angioplasty, involving specific tools to guarantee satisfying outcomes. This review aims to discuss the particular venous anatomy of the upper body, the physiopathology of superior vena cava obstruction, and specificities of endovascular treatment compared with other management options.

## Introduction

William Hunter first described superior vena cava (SVC) syndrome in 1757 as a complication of compression from an aortic aneurysm (1). Superior vena cava obstruction (SVCO) may complicate many mediastinal disorders whose etiologies have changed considerably in recent years. Until about 60 years ago, infectious diseases accounted for the majority of cases (particularly tuberculosis and syphilitic aortic aneurysm). Since then, malignant conditions have become the major cause followed by non-malignant thrombosis, reflecting the increased use of intravascular catheters and pacemakers leads (2, 3).

Limitation of venous blood return from the head, arms, and upper torso to the heart through the SVC results in a constellation of signs and symptoms, constituting the SVC syndrome. As in other parts of the body, blood flows through collateral vessels in case of vessel obstruction. In SVCO, collateral flow is directed to the lower body and inferior vena cava (IVC) or the azygos vein. The severity of symptoms depends on several factors, including the speed of onset, degree of narrowing, and the quality of collateral supply.

Imaging studies play a crucial role in the diagnosis and choice of treatment. Management is guided by the underlying etiology and the severity of symptoms.

This article aims to review the causes and endovascular therapeutic options for SVCO.

## SVCO Pathophysiology

### Relevant Anatomic Considerations

Vascular occlusion brings about collateral circulation. When the SVC blood flow is compromised, collaterals may form to shunt the flow back to the heart. Depending on the obstruction site, collaterals may be intra- or extrathoracic. The most important pathway is the azygos system—the latter consists of the azygos, hemiazygos, and accessory hemiazygos veins. Depending on the level of obstruction, the collateral network and, therefore, the symptoms, will vary (4).

SVC occlusion below the junction of the azygos vein arch will allow direct and indirect shunting by the azygos system. Direct shunting will pass countercurrent through the azygos system to the IVC. Indirect shunting will pass through the pericardo-phrenic veins and secondarily join the azygos system and the IVC (through esophageal, diaphragmatic, mediastinal, and bronchial veins). Transverse shunting also exists through communications from the azygos system to the intra- and extra-spinal venous plexuses. The superficial venous system also allows a cavo-caval anastomosis (through the internal thoracic veins and epigastric veins to the external iliac veins or through the external thoracic veins and superficial epigastric veins to the internal saphenous veins). Parietal veins may also join the para-umbilical veins.

Other shunting may form a right-to-left venous shunt (from anastomosis between the azygos system to the bronchial and then pulmonary veins) or a shunt to the portal system (esophageal veins to the left gastric veins or from anastomoses to the para-umbilical veins and ligamentum teres) (5, 6).

SVC occlusion above the azygos vein arch or the occlusion of the innominate vein on one side will force the development of supra-sternal venous anastomoses, allowing mainly a transverse contralateral shunt (anterior and external jugular veins are directly and indirectly anastomosed by the thyroid veins). Posterior anastomoses also exist with the vertebral veins and the spinal plexuses. By these derivations, blood will flow through the superior intercostal veins to the azygos system and the SVC below the occlusion.

Knowledge of this particular anatomy explains, therefore, why clinical SVC syndrome doesn't develop when only one brachiocephalic vein is occluded and also that the symptoms are usually less severe if the level of obstruction is below the azygos system.

A comprehensive classification of SVCO based on previsouly published works (7, 8) integrating anatomic patterns that may impact the treatment strategy is provided here:

- Type I—isolated stenosis of the superior vena cava
- Type II—stenosis of central veins (subclavian/brachio-cephalic) with or without extension to SVC
- Type III—chronic total occlusion of the SVC
- Type IV—chronic total occlusion of one or more central veins with or without extension to SVC
  - IVa: occlusion of the brachiocephalic veins
  - IVb: occlusion of the brachiocephalic veins and SVC
  - IVc: occlusion of brachiocephalic veins, SVC and jugular veins

### Etiologies of the SVCO

SVCO can be subdivided into benign or malignant and extrinsic or intrinsic etiologies (Table 1). Malignant causes are the most common (85% of cases) with bronchopulmonary cancer, mainly small cell cancers, germ cell tumors, and lymphoma as the most frequent. They induce mainly extrinsic compression of the central veins (9). Benign extrinsic causes are much rarer, such as benign tumors (goiter, teratoma), cardiovascular causes (aneurysm, constrictive pericarditis, pericardial effusion), and acute or chronic mediastinitis.

**Table 1:** SVCO etiologies.

**Etiologies**	
**Benign**	**Malignant**
External compression - Cardiovascular diseases - Lung diseases - Trauma - Mediastinitis - Thyroid goiter - Thymoma - Cystic hygroma Intraluminal fibrosis - Idiopathic - Long-term catheters - Pacemakers/defibrillators leads - Hypercoagulable state - Infectious (tuberculosis, syphilis, and histoplasmosis)	Lung cancer Lymphoma Sarcoma Metastatic cancer Leiomyosarcoma Plasmocytoma

*Most frequent etiologies of SVCO classified as benign and malignant*.

On the other hand, intrinsic causes are less common (3–20% of cases) but are the leading benign etiologies (9). In the vast majority of cases, benign causes involve a thrombus around a foreign body such as a central catheter or, more rarely, pacemaker wires. Venous thrombosis is favored either by endothelial lesions from mechanical friction against the vessel wall (for example, due to a short catheter tip above the right atrium) or rheological modifications caused by flow turbulences. Other favoring factors are related to the toxicity of the administered treatment (for example, chemotherapy through an implanted venous access device) or hypercoagulability of cancer patients (10, 11).

## Clinical Profile OF SVCO

The severity of symptoms depends on the degree of narrowing and speed of onset. History should always specify the duration of symptoms, previous diagnoses of malignant diseases, or previous intravascular interventions. Symptoms are usually progressive over a few weeks and may improve in some cases as collaterals develop.

Patients can present with a wide variety of symptoms, the most frequent being upper body swelling (face, neck, arms). Less commonly, chest pain, respiratory symptoms (edema of the larynx or pharynx causing cough, stridor, dyspnea, dysphagia, hoarseness), or neurologic manifestations (cerebral edema causing headaches, confusion, and possibly coma) can be observed (2, 3, 12–15).

There are two scoring systems to stratify the severity of superior vena cava syndrome (SVCS). Neither is validated, but both provide a valuable approach to select patients and create a decision tree. In the classification system proposed by Yu et al. (16), symptoms are categorized by their severity ranging from asymptomatic patients (grade 0) to severe (grade 3) and even life-threatening (grade 4) manifestations (Table 2), the two latter requiring emergent management. Grade 5 SVCS is lethal. The Kishi score (Table 3) also allows quantifying the gravity of symptoms and guide therapy. A score above 4 was retained as an indication for superior vena cava endoprosthesis by the authors (17).

**Table 2:** Yu Grading system (16).

**Grade**	**Category**	**Estimated incidence (%)**	**Definition**
0	Asymptomatic	10	Radiographic superior vena cava obstruction in the absence of symptoms
1	Mild	25	Edema in head or neck (vascular distention), cyanosis, plethora
2	Moderate	50	Edema in the head or neck with functional impairment (mild dysphagia, cough, mild or moderate impairment of head, jaw or eyelid movements, visual disturbances caused by ocular edema)
3	Severe	10	Mild or moderate cerebral edema (headache, dizziness) or mild/moderate laryngeal edema or diminished cardiac reserve (syncope after bending)
4	Life-threatening	5	Significant cerebral edema (confusion, obtundation) or significant laryngeal edema (stridor) or significant hemodynamic compromise (syncope without precipitating factors, hypotension, renal insufficiency)
5	Fatal	<1	Death

*Each sign or symptom must raise suspicion of superior vena cava obstruction and the effects of cerebral or laryngeal edema or impact on cardiac function. Symptoms caused by other factors (e.g., vocal cord paralysis, compromise of the tracheobronchial tree, or heart as a result of mass effect) should not be considered as they are due to mass effect on other organs and not superior vena cava obstruction*.

**Table 3:** Kishi Score (17).

**Signs and symptoms**	**Grade**
**Neurologic symptoms**	
- Stupor, coma, or blackout	4
- Blurry vision, headache, dizziness, or amnesia	3
- Changes in mentation	2
- Uneasiness	1
**Laryngopharyngeal or thoracic symptoms**	
- Orthopnea or laryngeal edema	3
- Stridor, hoarseness, dysphagia, glossal edema, or shortness of breath	2
- Cough or pleural effusions	1
**Nasal and facial signs or symptoms**	
- Lip edema, nasal stiffness, epistaxis, or rhinorrhea	2
- Facial swelling	1
**Venous dilatation**	
- Neck vein or arm vein distention, upper extremity swelling, or upper body plethora	1

*The scoring system of Kishi et al. (17), is used to quantify the gravity of symptoms. A score above 4 was retained as an indication for superior vena cava endoprosthesis by the authors. The total score for signs and symptoms was calculated as the sum of the highest grades in each class*.

Depending on how rapidly the symptoms appeared, SVCO can also be classified as acute, subacute, and chronic with a direct repercussion on treatment:

- *Acute*: <2 weeks, management relies mainly on thrombolysis and/or thromboaspiration and anticoagulation.
- *Subacute and chronic*: Subacute (2 weeks-2 months) or chronic (>2 months) SVCO, on the other hand, are best approached by endovascular therapies (angioplasty and/or stenting).

## SVCO Imaging

Diagnosis of venous obstruction requires two findings—first, reduced vessel opacification distal to the obstruction, intraluminal filling defects, or an occlusive lesion—second, the presence of collateral pathways. Phlebography, either catheter-based or by computed tomography (CT) provides the most expedient diagnosis (18). To guide the endovascular treatment, additional information provided only by CT, such as vein thickness and the vein wall's fibrotic pattern, is helpful.

Catheter-based phlebography provides the fastest path to both diagnosis and immediate treatment (endovascular recanalization), in case of severe or life-threatening symptoms (cerebral edema, laryngeal edema, and hemodynamic compromise).

On the other hand, CT phlebography may better determine the precise origin of SVCO and is suitable for treatment planning in patients with less severe symptoms. CT also helps define the level and extent of venous blockage as well as collateral pathways of venous drainage, but above all, it allows to identify the underlying cause of venous obstruction. CT phlebography must be performed with a specific protocol to reduce flow artifacts due to non-opacified veins (18).

As cross-sectional imaging, magnetic resonance imaging (MRI) may help in the same manner in patients with a known allergy to iodinated contrast or when venous access cannot be obtained for contrast-enhanced studies (19).

Duplex ultrasound can be used for peripheral analysis (subclavian, axillary, and brachiocephalic veins) and indirect findings that suggest SVCO (dampening of the venous waveforms in the upper body with loss of venous pulsatility and respiratory variation) but cannot image the SVC directly.

## Treatment Options

Guidelines for SVCS are lacking, but traditionally treatment approaches have included chemotherapy and/or radiation therapy, surgical bypass, or endovascular therapy. Case selection is, as always, crucial to offer the right treatment for each patient and decisions should account for the clinical course (acute/subacute/chronic), degree of urgency to treat the SVCS, chemosensitivity of the cancer when tumoral, and cross-sectional imaging results.

## Medical Treatment of SVCO

Medical management aims to relieve symptoms, preferably through relief of the obstruction but not always. A pure symptomatic benefit may be obtained by reducing hydrostatic pressure when elevating the patient's head or administering loop diuretics. The real effect of those maneuvres is, however, debated. In the same way, glucocorticoids are often prescribed in SVCO of malignant origin, while their effect is also questionable (2). Still, in malignant causes, SVCO may be resolved with medical therapy if the tumor's histology is predictive of good tumor response (small cell carcinoma, lymphoma, and germ cell tumors). Chemotherapy or radiotherapy may induce a rapid improvement of symptoms, however not immediate (2, 14, 20). Symptoms may even be transiently worse due to the inflammatory phase of treatment. In benign causes, most often due to intravascular devices, removing the catheter and anticoagulation therapy may be beneficial. Surgical bypass with venous graft may also retain some indications, mostly in benign cases, but is often substituted by endovascular treatment, as surgery is associated with more complications and longer hospital stays than endovascular therapies (21, 22).

## Strategies for Endovascular Therapies

Acute SVCO management relies mainly on thrombolysis and/or thrombo-aspiration combined with anticoagulation for inpatients. Conversely, subacute and chronic SVCO is best treated by angioplasty and/or stenting. The latter are also commonly performed for inpatients. However, depending on the complexity, underlying disease, and co-morbidities, some procedures might be done on a day care basis.

### Principle of Angioplasty

Endovascular treatment is usually performed under fluoroscopic guidance. Ultrasound is used to guide the percutaneous puncture of veins. Rarely, additional techniques might be used, such as intravascular ultrasound, cone-beam CT, or transesophageal echography.

Procedures can be performed under local anesthesia in simple cases, but general anesthesia is preferred when multiple accesses are necessary (typically type III and IV requiring recanalization of the occlusion).

Venous access can be unique or combined (femoral, basilic, and/or internal jugular). The typical introducer sheath is 10F 60 cm to allow insertion of large diameter stents from the femoral veins. In the case of double access, an additional 6F is usually sufficient. 5,000 to 10,000 Units (UI) of heparin are administered. Perfusion of sheaths with heparin infusion is also performed.

The standard angioplasty technique using a balloon over a wire extensively described in the literature for different anatomic sites is also valid for the SVCO. Hydrophilic or hybrid guidewires are most commonly used to cross the stenosis or occluded veins. Stiffer guidewires are necessary for large diameter stents (>14 mm). In chronic total occlusions (CTO) or severe stenosis due to implanted metallic devices, guidewires are supported by dedicated catheters (CTO catheters) to give the necessary push for stenosis/CTO crossing. CTO wires on 0.018 are rarely used.

Venous CTO requires gradual balloon angioplasty with small balloons first and then larger ones to overcome venous stricture. High-pressure balloons are preferred. In a CTO with complete fibrotic stenosis, a scoring balloon might help to open the vessel.

After angioplasty, stents are used. Self-expandable stents are preferred—oversized by 10–20% in diameter and length adjusted to the obstruction span. Multiple overlapped stents can be delivered if necessary. In-stent remodeling, if necessary, is performed with shorter in-stent balloon dilatation, with caution paid to avoid stent displacement. A long introducer sheath (60 cm) helps secure balloon retrieval. To prevent secondary sent migration, the objective is to restore sufficient flow and not necessarily reach 0% residual stenosis. A mild non-hemodynamically significant stricture in the stent is usually considered optimal.

Self-expanding (nitinol, elgiloy), balloon-expandable (cobalt chromium, stainless steel) as well as self-expanding covered (PTFE-coated nitinol) stents and stent-grafts have been used with similar results (23–33). However, self-expanding stents with greater flexibility may be advantageous for adaptability and ease of positioning and deployment (27). On the other hand, covered stents may offer better long-term patency but without any significant effect on clinical success rate or survival (30, 34). Covered stents should also be used with caution due to concerns of migration and coverage of venous collaterals.

Adjunctive therapies, such as thrombectomy or catheter-directed thrombolysis, are used in the case of associated thrombotic material.

Sometimes, thrombectomy must be applied to dissolve associated large clots above the obstruction.

### Endpoints of SVO Revascularization

**Figure 1:**
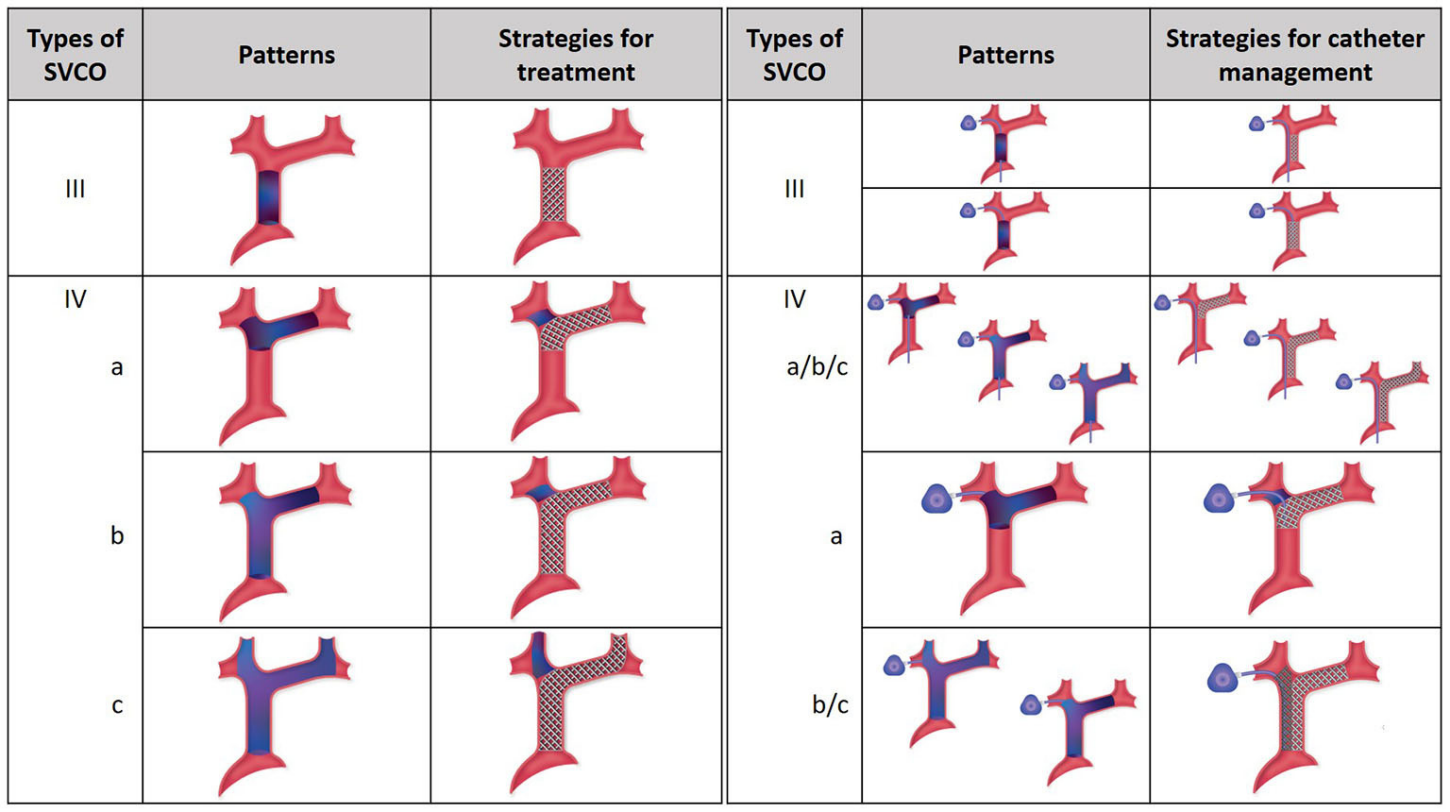
Management of chronic venous occlusion with and without central venous catheters. On the left, SVCO with CTO (type III and IV) are illustrated with their respective strategies for treatment. Depending on the extension of the occlusion from the central veins to the superior vena cava, three types are separated. The aim for treatment is recanalization and stenting extending if necessary to the dominant jugular axis. On the right, strategies for central venous catheter management are illustrated. In type III SVCO with long catheter, direct stenting is done without repositioning of the catheter, which is flattened between the venous wall and the stent, the tip is still free and functional at the cavoatrial junction. In type III SVCO with short catheter, the tip of the catheter is repositioned inside the stent lumen after angioplasty to keep it functional. In type IV SVCO with long catheters, whatever the subtypes, recanalization and stenting are managed without repositioning of the tip, which is still free and functional at the cavoatrial junction. In type IVa SVCO with short catheter, the tip is repositioned inside the stent through the stent mesh. In type IVb and IVc SVCO with short catheters, double stenting is necessary with repositioning of the tip of the catheter inside the ipsilateral stent. SVCO, Superior Vena Cava Obstruction; CTO, Complete Total Occlusion.

For type I and II, treatment can be limited to the site of stenosis. For type III and IV, the goal is to re-establish direct venous flow between the heart and the brain through the dominant jugular axis. If not possible, as is sometimes the case in type IV, the non-dominant jugular vein is treated. As a last resort, if no jugular axis can be re-vascularized, flow is re-established between the SVC and a subclavian vein. Figure 1 illustrates the goals of venous reconstructions in various cases of chronic total occlusion without and with central venous catheters.

### Types of SVC Reconstruction

Different reconstructions are possible in the SVC system. One line reconstruction, defined as revascularization based on connecting the dominant jugular vein to the proximal SVC or even the right atrium, is the most appropriate strategy to resolve SVC syndrome. Two line reconstruction aims to reconstruct both sides (brachiocephalic or jugular veins) to the proximal SVC based on a Y reconstruction or kissing stents into the SVC. This reconstruction is useful for type IV CTO in patients who need a central venous catheter through the stent. In such cases, we prefer asymmetric reconstruction as previously reported (35). Bilateral symmetric reconstruction was reported to have no clinical benefit and is associated with higher complication rates and recurrence of SVCO (31).

### Management of Implanted Central Venous Catheters During SVCO Revascularization

**Figure 2:**
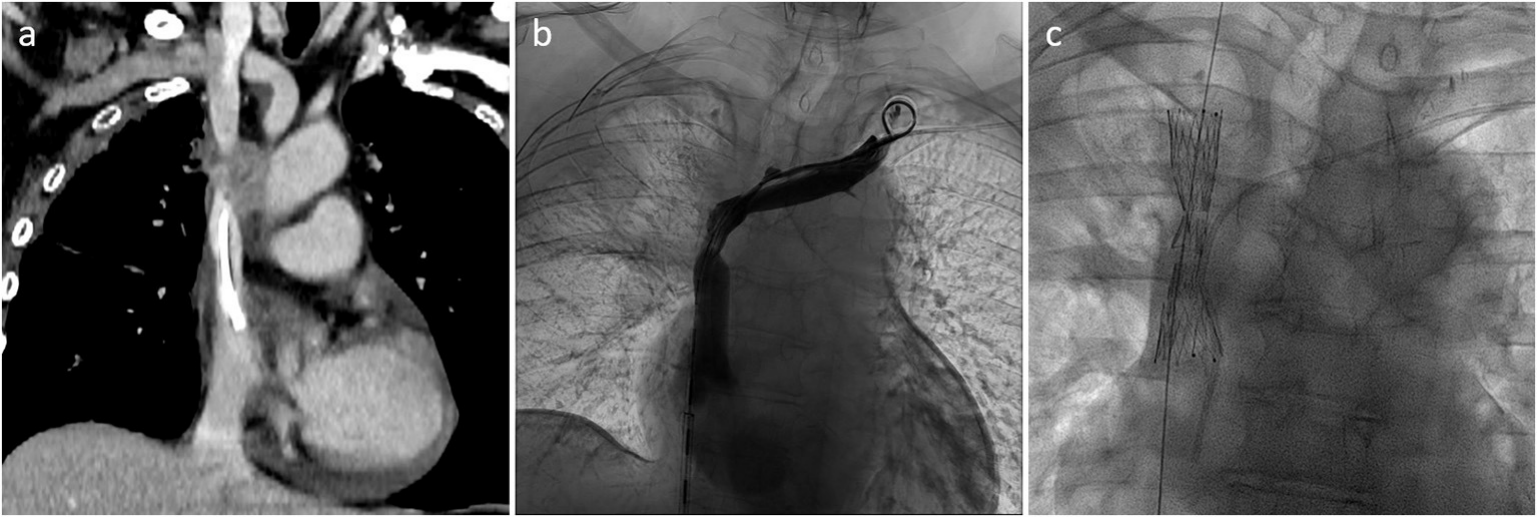
**(a,b)** Type I SVCO in a patient with a well-positioned implanted central venous catheter. **(c)** Stenting of the SVC was performed without repositioning of the catheter tip which ends below the stent.

**Figure 3:**
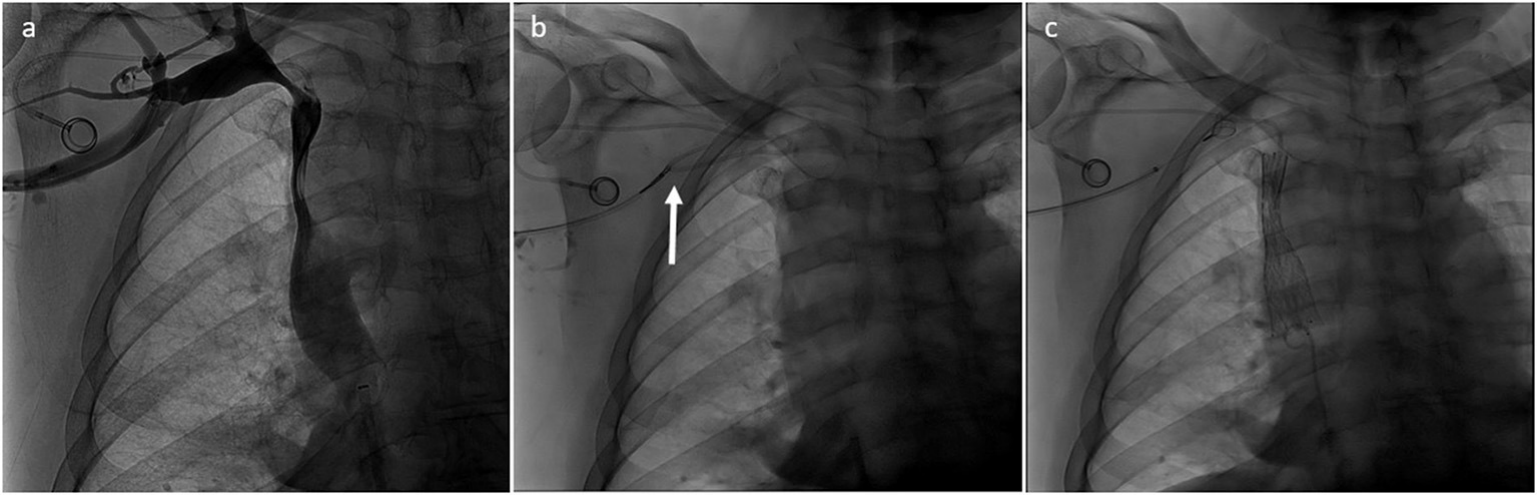
**(a)** Type II SVCO in patient with type III malposition of the tip of the implanted central venous catheter. Left jugular vein was dominant. **(b)** Tip of the central catheter was withdrawn from the superior vena cava with a snare inserted by the right arm (arrow). **(c)** Superior vena cava was treated and finally the tip of the implanted central venous catheter was repositioned in the SVC inside the stent lumen.

**Figure 4:**
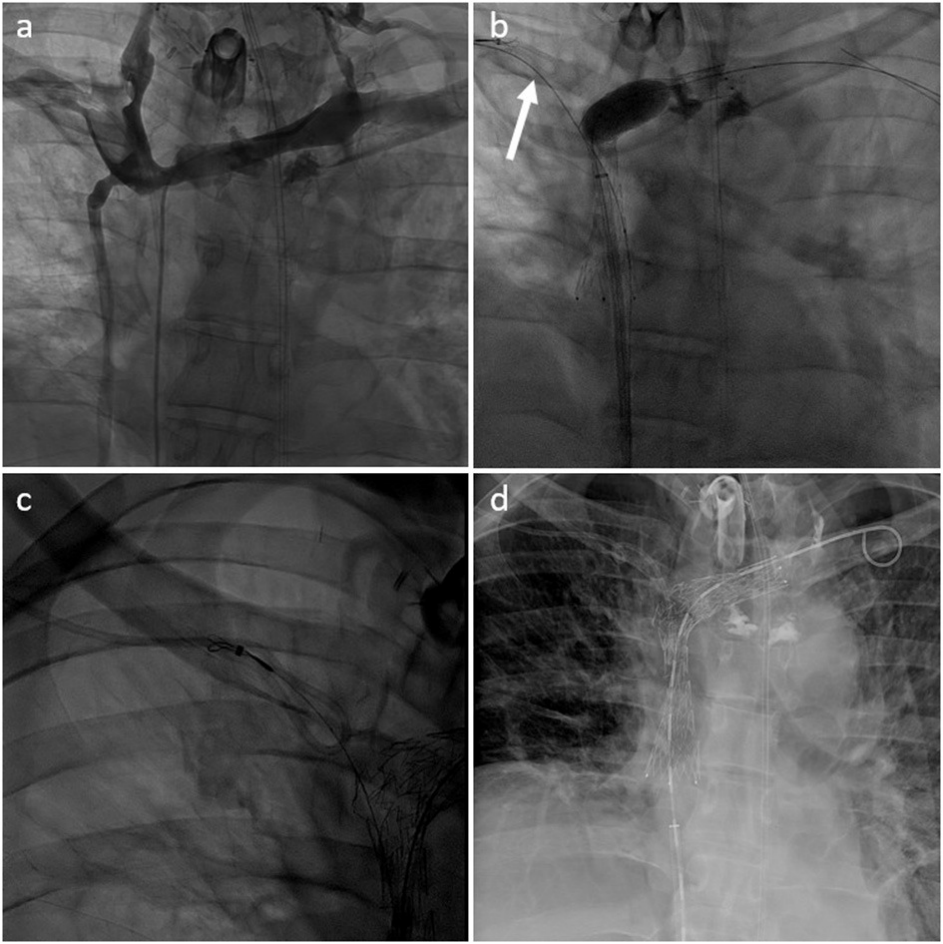
**(a)** Type IV SVCO with occlusion of the superior vena cava associated with stenosis of both innominate veins (type IV) in a patient with a left dominant jugular axis and a short implanted central venous catheter. **(b)** Central venous catheter was withdrawn from the superior vena cava with a snare inserted through the right arm (arrow). Recanalization and angioplasty of the superior vena cava and left innominated was performed with implantation of two overlapping stents. **(c)** Tip of the venous catheter was snared from the femoral venous access through the mesh of the SVC stent to allow repositioning. **(d)** Final result with the tip of the central venous catheter ending inside the SVC stent lumen.

Preserving previously implanted central venous catheters can be challenging when treating SVCO. First of all, tip positioning must be precisely determined. According to Glauser et al., three types are distinguished (36). In type I positioning, the tip is located more than or <1 cm from the superior cavoatrial junction (CAJ). Endovascular reconstruction of the superior vena cava is usually possible without mobilization of the catheter. The catheter will be flattened between the stent wall and the vessel, but the tip will stay free at the cavoatrial junction, saving device function. On the contrary, in some type II or III positioning, the catheter tip is suboptimal or non-optimal. In type II positioning, the tip is located >1 cm above or below the cavoatrial junction. In type III, the tip is located >3 cm below the CAJ or not inside the SVC. Stenting at the tip level may render the catheter unusable (type II or III with the tip above the CAJ). In such cases, the catheter should be withdrawn from the venous segment treated before endovascular reconstruction and then repositioned in the stent lumen. This maneuver is typically performed by brachial access using a snare to catch and mobilize the tip (35, 37). Figures 2–4 show examples of treatments. Sometimes, a supplemental manoeuver of mechanical adhesiolysis is required to allow catheter mobilization (38).

### Post-operative Care

Patients with significant pre-existing heart disease (ejection fraction <30%) might benefit from intermediate care facilities for continuous monitoring during 24 h following the SVCO revascularization, to prevent pulmonary edema. This is specially indicated for patients with subacute severe type III/IV SVCO. Although no consensus exists, at least a short-term anticoagulation or antiplatelet therapy is indicated (39). We recommend long-term anticoagulation therapy in case of residual stenosis and low hemorrhagic risk.

## Complications

The primary risk during venous angioplasty is vessel rupture, which may precipitate pericardial tamponade. Fagedet et al. found that using stents over 16 mm was the only significant predictive factor for complications (hemorrhage, acute pulmonary edema from SVC reopening, stent collapse, stent migration, stent infection, and death). The same authors found that the median time to recurrence was 30 days, and the risk factors were initial CTO of the SVC, initial thrombosis, and the use of steel stents.

Interestingly, the use of long-term anticoagulation therapy was not associated with changes in recurrence or complication rates (27). Finally, the risk of pulmonary edema can be prevented by close monitoring of patients with reduced cardiac function.

## Follow-Up

Generally, successful endovascular therapy ensues in more than 95% of patients, and over 90% of them report symptom relief (8, 24, 26, 39–41).

Restenosis (defined as occlusion or stent thrombosis) is infrequent, reported in about 13% of patients, and often, patency can be restored with re-intervention (39).

Independently of intervention success, the average life span in patients with malignant related SVCS is poor, about 6 months, but offering an immediate and sustained symptomatic relief (28).

On the other hand, survival is expected to be normal in patients with benign causes of SCVS, and endovascular stenting was shown to offer durable mid-term relief, equal to surgical reconstruction (22).

## Conclusions

Despite the absence of consensus, careful selection of patients with SCVS according to the etiology, severity, and length of symptoms may offer the best treatment with the highest likelihood of success. Endovascular treatment of SVCS is an effective and safe method to rapidly improve symptoms due to the obstruction of blood flow through the SVC.

## Author Contributions

AP wrote the first draft of the manuscript. AP, SS, DR, and SQ contributed to manuscript extension, revision, read, and approved the submitted version. All authors contributed to the article and approved the submitted version.

## Conflict of Interest

The authors declare that the research was conducted in the absence of any commercial or financial relationships that could be construed as a potential conflict of interest.

## Publisher's Note

All claims expressed in this article are solely those of the authors and do not necessarily represent those of their affiliated organizations, or those of the publisher, the editors and the reviewers. Any product that may be evaluated in this article, or claim that may be made by its manufacturer, is not guaranteed or endorsed by the publisher.

## Abbreviations

SVC: superior vena cava
SVCO: superior vena cava obstruction
IVC: inferior vena cava
MRI: magnetic resonance imaging
CT: computed tomography
CTO: chronic total occlusion
CAJ: cavoatrial junction.

## References

[1] Hunter W Johnston W. The History of an Aneurysm of the Aorta, with Some Remarks on Aneurysms in General. London: William Johnston (1757).

[2] Wilson LD Detterbeck FC Yahalom J. Clinical practice. Superior vena cava syndrome with malignant causes. N Engl J Med. (2007) 356:1862–9. 10.1056/NEJMcp067190 17476012

[3] Rice TW Rodriguez RM Light RW. The superior vena cava syndrome: clinical characteristics and evolving etiology. Medicine. (2006) 85:37–42. 10.1097/01.md.0000198474.99876.f0 16523051

[4] Lacout A Marcy PY Thariat J Lacombe P El Hajjam M. Radio-anatomy of the superior vena cava syndrome and therapeutic orientations. Diagn Interv Imaging. (2012) 93:569–77. 10.1016/j.diii.2012.03.025 22560123

[5] Ho HT Horowitz AL Ho AC. Systemic to pulmonary venous communication (right-to-left shunt) in superior vena cava obstruction demonstrated by spiral CT. Br J Radiol. (1999) 72:712–3. 10.1259/bjr.72.859.10624332 10624332

[6] Dickson AM. The focal hepatic hot spot sign. Radiology. (2005) 237:647–8. 10.1148/radiol.2372031690 16244273

[7] Stanford W Jolles H Ell S Chiu LC. Superior vena cava obstruction: a venographic classification. AJR Am J Roentgenol. (1987) 148:259–62. 10.2214/ajr.148.2.259 3492099

[8] Breault S Doenz F Jouannic AM Qanadli SD. Percutaneous endovascular management of chronic superior vena cava syndrome of benign causes: long-term follow-up. Eur Radiol. (2017) 27:97–104. 10.1007/s00330-016-4354-y 27085696

[9] Nieto AF Doty DB. Superior vena cava obstruction: clinical syndrome, etiology, and treatment. Curr Probl Cancer. (1986) 10:441–84. 10.1016/S0147-0272(86)80006-X 3757550

[10] Qanadli SD El Hajjam M Mignon F de Kerviler E Rocha P Barré O. Subacute and chronic benign superior vena cava obstructions: endovascular treatment with self-expanding metallic stents. AJR Am J Roentgenol. (1999) 173:159–64. 10.2214/ajr.173.1.10397119 10397119

[11] Teo N Sabharwal T Rowland E Curry P Adam A. Treatment of superior vena cava obstruction secondary to pacemaker wires with balloon venoplasty and insertion of metallic stents. Eur Heart J. (2002) 23:1465–70. 10.1053/euhj.2002.3260 12208227

[12] Yellin A Rosen A Reichert N Lieberman Y. Superior vena cava syndrome. The myth–the facts. Am Rev Respir Dis. (1990) 141(5 Pt 1):1114–8. 10.1164/ajrccm/141.5_Pt_1.1114 2339833

[13] Schraufnagel DE Hill R Leech JA Pare JA. Superior vena caval obstruction. Is it a medical emergency? Am J Med. (1981) 70:1169–74. 10.1016/0002-9343(81)90823-8 7234887

[14] Armstrong BA Perez CA Simpson JR Hederman MA. Role of irradiation in the management of superior vena cava syndrome. Int J Radiat Oncol Biol Phys. (1987) 13:531–9. 10.1016/0360-3016(87)90068-X 3558044

[15] Markman M. Diagnosis and management of superior vena cava syndrome. Cleve Clin J Med. (1999) 66:59–61. 10.3949/ccjm.66.1.59 9926632

[16] Yu JB Wilson LD Detterbeck FC. Superior vena cava syndrome–a proposed classification system and algorithm for management. J Thorac Oncol. (2008) 3:811–4. 10.1097/JTO.0b013e3181804791 18670297

[17] Kishi K Sonomura T Mitsuzane K Nishida N Yang RJ Sato M. Self-expandable metallic stent therapy for superior vena cava syndrome: clinical observations. Radiology. (1993) 189:531–5. 10.1148/radiology.189.2.8210386 8210386

[18] Qanadli SD El Hajjam M Bruckert F Judet O Barré O Chagnon S. Helical CT phlebography of the superior vena cava: diagnosis and evaluation of venous obstruction. AJR Am J Roentgenol. (1999) 172:1327–33. 10.2214/ajr.172.5.10227511 10227511

[19] Sonavane SK Milner DM Singh SP Abdel Aal AK Shahir KS Chaturvedi A. Comprehensive imaging review of the superior vena cava. Radiographics. (2015) 35:1873–92. 10.1148/rg.2015150056 26452112

[20] Spiro SG Shah S Harper PG Tobias JS Geddes DM Souhami RL. Treatment of obstruction of the superior vena cava by combination chemotherapy with and without irradiation in small-cell carcinoma of the bronchus. Thorax. (1983) 38:501–5. 10.1136/thx.38.7.501 6310812 PMC459595

[21] Doty JR Flores JH Doty DB. Superior vena cava obstruction: bypass using spiral vein graft. Ann Thorac Surg. (1999) 67:1111–6. 10.1016/S0003-4975(99)00145-9 10320259

[22] Rizvi AZ Kalra M Bjarnason H Bower TC Schleck C Gloviczki P. Benign superior vena cava syndrome: stenting is now the first line of treatment. J Vasc Surg. (2008) 47:372–80. 10.1016/j.jvs.2007.09.071 18241760

[23] Garcia Monaco R Bertoni H Pallota G Lastiri R Varela M Beveraggi EM. Use of self-expanding vascular endoprostheses in superior vena cava syndrome. Eur J Cardiothorac Surg. (2003) 24:208–11. 10.1016/S1010-7940(03)00293-8 12895609

[24] Nagata T Makutani S Uchida H Kichikawa K Maeda M Yoshioka T. Follow-up results of 71 patients undergoing metallic stent placement for the treatment of a malignant obstruction of the superior vena cava. Cardiovasc Intervent Radiol. (2007) 30:959–67. 10.1007/s00270-007-9088-4 17546400

[25] Kee ST Kinoshita L Razavi MK Nyman UR Semba CP Dake MD. Superior vena cava syndrome: treatment with catheter-directed thrombolysis and endovascular stent placement. Radiology. (1998) 206:187–93. 10.1148/radiology.206.1.9423671 9423671

[26] Urruticoechea A Mesia R Dominguez J Falo C Escalante E Montes A. Treatment of malignant superior vena cava syndrome by endovascular stent insertion. Experience on 52 patients with lung cancer. Lung Cancer. (2004) 43:209–14. 10.1016/S0169-5002(03)00361-1 14739042

[27] Fagedet D Thony F Timsit JF Rodiere M Monnin-Bares V Ferretti GR. Endovascular treatment of malignant superior vena cava syndrome: results and predictive factors of clinical efficacy. Cardiovasc Intervent Radiol. (2013) 36:140–9. 10.1007/s00270-011-0310-z 22146975

[28] Sobrinho G Aguiar P. Stent placement for the treatment of malignant superior vena cava syndrome - a single-center series of 56 patients. Arch Bronconeumol. (2014) 50:135–40. 10.1016/j.arbr.2014.03.001 24360084

[29] Bialkowski J Szkutnik M Fiszer R Glowacki J Zembala M. Percutaneous dilatation of coarctation of the aorta, stenotic pulmonary arteries or homografts, and stenotic superior vena cava using Andrastents XL and XXL. Kardiol Pol. (2011) 69:1213–9. Available online at: https://journals.viamedica.pl/kardiologia_polska/article/view/79442 22219090

[30] Gwon DI Ko GY Kim JH Shin JH Yoon HK Sung KB. Malignant superior vena cava syndrome: a comparative cohort study of treatment with covered stents versus uncovered stents. Radiology. (2013) 266:979–87. 10.1148/radiol.12120517 23249571

[31] Dinkel HP Mettke B Schmid F Baumgartner I Triller J Do DD. Endovascular treatment of malignant superior vena cava syndrome: is bilateral wallstent placement superior to unilateral placement? J Endovasc Ther. (2003) 10:788–97. 10.1177/152660280301000416 14533962

[32] Nguyen NP Borok TL Welsh J Vinh-Hung V. Safety and effectiveness of vascular endoprosthesis for malignant superior vena cava syndrome. Thorax. (2009) 64:174–8. 10.1136/thx.2007.086017 19176843

[33] Lanciego C Pangua C Chacon JI Velasco J Boy RC Viana A. Endovascular stenting as the first step in the overall management of malignant superior vena cava syndrome. AJR Am J Roentgenol. (2009) 193:549–58. 10.2214/AJR.08.1904 19620456

[34] Quaretti P Galli F Moramarco LP Corti R Leati G Fiorina I. Stent grafts provided superior primary patency for central venous stenosis treatment in comparison with angioplasty and bare metal stent: a retrospective single center study on 70 hemodialysis patients. Vasc Endovascular Surg. (2016) 50:221–30. 10.1177/1538574416639149 27097842

[35] Volpi S Doenz F Qanadli SD. Superior vena cava (SVC) endovascular reconstruction with implanted central venous catheter repositioning for treatment of malignant SVC obstruction. Front Surg. (2018) 5:4. 10.3389/fsurg.2018.00004 29435452 PMC5790922

[36] Glauser F Breault S Rigamonti F Sotiriadis C Jouannic AM Qanadli SD. Tip malposition of peripherally inserted central catheters: a prospective randomized controlled trial to compare bedside insertion to fluoroscopically guided placement. Eur Radiol. (2017) 27:2843–9. 10.1007/s00330-016-4666-y 27957644

[37] Qanadli SD Mesurolle B Sissakian JF Chagnon S Lacombe P. Implanted central venous catheter-related acute superior vena cava syndrome: management by metallic stent and endovascular repositioning of the catheter tip. Eur Radiol. (2000) 10:1329–31. 10.1007/s003300000361 10939501

[38] Breault S Glauser F Babaker M Doenz F Qanadli SD. Percutaneous endovascular salvage techniques for implanted venous access device dysfunction. Cardiovasc Intervent Radiol. (2015) 38:642–50. 10.1007/s00270-014-0968-0 25192947

[39] Uberoi R. Quality assurance guidelines for superior vena cava stenting in malignant disease. Cardiovasc Intervent Radiol. (2006) 29:319–22. 10.1007/s00270-005-0284-9 16502166

[40] Baltayiannis N Magoulas D Anagnostopoulos D Bolanos N Sfyridis P Georgiannakis E. Percutaneous stent placement in malignant cases of superior vena cava syndrome. J BUON. (2005) 10:377–80. Available online at: https://jbuon.com/archive/10-3-377.pdf 17357192

[41] Courtheoux P Alkofer B Al Refaï M Gervais R Le Rochais JP Icard P. Stent placement in superior vena cava syndrome. Ann Thorac Surg. (2003) 75:158–61. 10.1016/S0003-4975(02)04293-5 12537210

